# Antioxidant Effects of Essential Oils from the Peels of Citrus Cultivars

**DOI:** 10.3390/molecules30040833

**Published:** 2025-02-11

**Authors:** Jiyoon Yang, Mi-Jin Park

**Affiliations:** 1 Division of Wood Industry, Department of Forest Products and Industry, National Institute of Forest Science, Seoul 02455, Republic of Korea; dldh89@korea.kr; 2 Division of Forest Industrial Materials, Department of Forest Products and Industry, National Institute of Forest Science, Seoul 02455, Republic of Korea

**Keywords:** citrus, essential oil, antioxidation, terpene, active compound

## Abstract

Essential oils from citrus cultivars are widely used in food, cosmetic, and pharmaceutical industries, and they have been extensively studied in the last decades. This study investigates the antioxidant activities of essential oils from 21 citrus cultivars and the active antioxidant constituents of the oils. Essential oils are extracted from the peels of citrus cultivars via hydrodistillation, and their chemical compositions are analyzed by gas-chromatography–mass-spectroscopy. The antioxidant activities of the citrus cultivars are determined using 2,2-diphenyl-1-picrylhydrazyl (DPPH), 2,2′-azino-bis(3-ethylbenzothiazoline-6-sulfonic acid (ABTS), and ferric-reducing antioxidant potential (FRAP) assays. Based on the results, the major constituent of the oils is d-limonene (50.88–97.19%). The essential oil from *Citrus junos* shows the highest phenolic content (360.04 ± 24.75 mg GAE/100 g), followed by that from *Citrus* × *latifolia* (339.42 ± 31.14 mg GAE/100 g), [(*Citrus unshiu* × *Citrus sinensis*) × *Citrus reticulata*] × *Citrus reticulata* (327.05 ± 14.29 mg GAE/100 g), and [(*Citrus unshiu* × *Citrus sinensis*) × *Citrus reticulata*] × *Citrus reticulata* (322.92 ± 21.43 mg GAE/100 g). The essential oil from [(*Citrus unshiu* × *Citrus sinensis*) × *Citrus reticulata*] × *Citrus reticulata* shows the highest DPPH and ABTS radical scavenging activity, with an EC_50_ of 86.17 ± 4.87 and 0.16 ± 0.06 mg/mL, respectively. The essential oil from *Citrus reticulata* and [(*Citrus unshiu* × *Citrus sinensis*) × *Citrus reticulata*] × *Citrus reticulata* shows the highest ferric-reducing activities (2302.55 ± 237.26 and 2213.12 ± 35.54 mg/100 g, respectively). These results indicate that the essential oil from [(*Citrus unshiu* × *Citrus sinensis*) × *Citrus reticulata*] × *Citrus reticulata* has a higher antioxidation effect than that from other cultivars. By comparing the chemical compositions of the essential oils, 12 compounds are selected as the major contributors to the antioxidant activities of the oils, and α-phellandrene and α-terpinene are the most active constituents of the oils.

## 1. Introduction

Oxidative stress is a phenomenon associated with the pathogenetic mechanisms of chronic diseases, such as diabetes mellitus, cancer, neurodegenerative diseases, and inflammatory diseases [1]. It is the imbalance between free-radical generation and radical scavenging systems [2]. Oxidative stress induces excessive production of ROSs, and lipids present in plasma, mitochondria, and endoplasmic reticulum membranes are the main targets of ROS attacks and peroxidation in most macromolecules [3]. ROSs are of a highly reactive nature due to unshared electron pairs in the molecular structure. Because of these structural characteristics, ROS react with intracellular biological macromolecules such as carbohydrates, nucleic acids, lipids, and proteins to alter their functions [4]. Most reactive oxygen species (ROSs), such as hydroxyl radical (•OH), hydrogen peroxide (H_2_O_2_), superoxide anion (O_2_^−^), and organic peroxides, are generated in cells by mitochondrial respiratory chains [5]. The hydroxyl radical (•OH) is highly reactive and causes severe damage to biomolecules. In addition, ROSs induce various kinase signaling pathways, resulting in chromatin modification such as histone acetylation/deacetylation and etc., which affect the expression of several genes [6]. As a result, excessive ROSs peroxidize lipids and causes cell damage, especially DNA damage; therefore, ROS regulation is essential for normal biological function [7]. Thus, there is a need for an •OH scavenger that prevents several diseases in the pharmaceutical field [8]. To that effect, several antioxidants have been investigated.

Butylated hydroxytoluene (BHT) and butylated hydroxyanisole (BHA) are widely used as chemical antioxidants [9]. However, they have side effects on humans and must be used in the smallest possible amount to maintain the antioxidation effect [10]. In Jayalakshmi’s study, BHT and BHA were administered to the blood of healthy rats, and, within 30 min, hemolytic activities were over 50% [11]. This indicates that BHA and BHT are harmful to the blood at a concentration of 0.75%. Although BHA and BHT are known to be metabolized in the liver and excreted through urine, they can be very harmful to the circulatory system; therefore, caution is required when using them. The global demand for healthy and safe products is increasing, and there is a need for novel industrial techniques to reduce the use of chemical additives and replace them with natural ones [12,13,14]. In addition to reducing the incidence of several diseases and improving flavor, this could extend the shelf life of foods.

Natural antioxidants can be found in plant parts, such as the leaves, fruits, and seeds [15,16]. Essential oils are functional substances with good antioxidation effects [17]. Essential oils, the second metabolite of plants, have been biosynthesized via a mevalonate pathway and are mainly composed of monoterpenes and sesquiterpenes [18]. Various essential oils have been investigated for their antioxidant and radical scavenging properties. The essential oil of *Eugenia caryophyllus* (IC_50_ 0.38 g/L) is the most potent scavenger of 2,2-diphenyl-1-picrylhydrazyl (DPPH) free radicals, even better than the standards ascorbic acid (IC_50_ 0.42 g/L) and BHT (IC_50_ 0.53 g/L) [19]. Considering the free radical scavenging activity, essential oils from *Mentha* × *piperita* L. and *Boswellia carterii* Birdwood also exhibit high 2,2′-azino-bis(3-ethylbenzothiazoline-6-sulfonic acid (ABTS) scavenging activity [20].

The genus *Citrus* belongs to the Rutaceae family, which comprises approximately 140 genera and 1300 species [21]. Citrus fruits are well-accepted worldwide because of their attractive color, pleasant aroma, and flavor [22]. They contain various biologically active compounds, such as phytochemicals, which have health benefits. Thus, the contribution of citrus species to various physiological activities has been studied [23]. More than 170 antioxidant compounds from citrus fruits, including vitamins, phenolic compounds, pectin, mineral elements, and terpenoids, have been reported [24]. One way to control inflammation is to limit oxidative stress. Citrus cultivars are rich in vitamin C and folate, both of which play a role in sustaining the integrity of the immune barrier and supporting the function of immune cells [25]. In summary, various biologically active compounds in citrus cultivars control oxidative stress and inflammation and support innate and acquired immune responses. In addition, the polyphenol fraction derived from citrus fruit is known to have antiglycation and digestive enzyme inhibition properties [26]. Citrus plants are a major source of essential oils, which have been extensively studied for their potential uses in the food industry [27]. The main component of essential oils extracted from citrus cultivars is limonene (32–98%) [28]. Previous studies have shown that limonene has anti-inflammatory, antioxidant, antinociceptive, and anticancer effects [29]. In addition, γ-terpinene, linalool, linalyl acetate, α-terpineol, (*E*)-β-ocimene, terpinolene, and β-pinene are also known as the main volatile components of citrus essential oils [30]. Due to these components of citrus essential oils, studies are being conducted to apply them to food preservatives [31]. However, some citrus cultivars produce essential oils by expression, which can produce non-volatile components such as bergamottin, isopimpinellin, and citropten, all of which can cause photosensitivity and skin irritation [32]. Therefore, extensive research should be conducted to ensure the safe use of citrus fruits.

The antioxidant activity of essential oils extracted from different species of citrus cultivars has been investigated. Mahmoud investigated the antioxidant activity of the essential oils from three citrus cultivars (*C. aurantifolia*, *C. limon*, and *C. paradisi*) using DPPH and β-carotene-linoleic acid assays [33]. The essential oil of *C. paradisi* showed the highest antioxidant activity. The essential oils of *C. latifolia*, *C. limon*, and *C. reticulata* also exhibit good radical scavenging ability compared to those of other citrus cultivars [34,35,36]. However, the antioxidant activity of only a limited citrus species has been studied.

This study evaluates the antioxidant activities of essential oils extracted from the peels of 21 citrus cultivars. Among them, the cultivar with the highest activity is determined, and the active antioxidant constituents that contribute to the antioxidant activity are identified.

## 2. Materials and Methods

### 2.1. Plant Materials

The peels of 21 ripened citrus cultivars were collected at two GPS locations in Jeju Island from 2 to 3 December 2019: four from the tangerine orchard (N 33°17′27.70″, E 126°41′38.70″) and 17 from the National Institute of Horticultural and Herbal Science (N 33°18′05.60″, E 126°36′43.60″). The data of the samples are listed in Table 1. The samples were kept in the National Institute of Forest Science’s herbarium.

### 2.2. Extraction of Essential Oils

Essential oils were extracted from the citrus cultivar peels via hydrodistillation using a Clevenger-type apparatus [37]. In brief, a 10 L round-bottom flask containing 1.0 kg of the citrus cultivar peels was placed in a digital heating mantle (MS-DM 608, MTOPS^®^, Yangju, Republic of Korea) and 6.0 L of distilled water was added and allowed to stand for more than 30 h at 102 ± 3 °C. The essential oils obtained were dried under anhydrous sodium sulfate (SAMCHUN, Seoul, Republic of Korea, 98.5%), transferred to dark vials, and then stored in the refrigerator at 4 °C until use.

### 2.3. Gas-Chromatography–Mass-Spectroscopy Analysis

The volatile constituents of the essential oils were analyzed by gas-chromatography–mass-spectroscopy (GC–MS; Trace 1310/ISQ-LT, ThermoScientific, Waltham, MA, USA) equipped with a VF-5MS capillary column (60 m × 0.25 mm, 0.25 μm; Agilent Technologies, Inc., Santa Clara, CA, USA). The temperature of the GC injector was set to 250 °C, and the flow rate of the helium carrier gas was 1.0 mL/min. The initial oven temperature was 50 °C (5 min), followed by a temperature increase to 65 °C (30 min) at 10 °C/min. Thereafter, the temperature was then raised to 210 °C (10 min) at 5 °C/min and, finally, to 325 °C (10 min) at 20 °C/min. The MS was conducted in the electron ionization mode. The ion source and interface temperature were set to 270 °C and 250 °C, respectively, and a mass range of 35–550 amu was recorded in the full scan mode. The Kovats retention index (KI) of the individual compounds was evaluated by comparing their relative retention times with those of an *n*-alkanes mixture (C_8_–C_30_, Sigma-Aldrich, St. Louis, MO, USA) in a VF-5MS column. The volatile constituents were identified by comparing their calculated KIs with the reported values (e.g., NIST Chemistry WebBook).

### 2.4. Determination of the Total Phenolic Content

The total phenolic content (TPC) of the essential oils was determined using the Folin–Ciocalteu method, and the experimental conditions were modified to suit the environment based on Fernandes’ paper [38]. The essential oils (500 μL) were mixed with 2.5 mL of 2 N Folin–Ciocalteu reagent (Sigma-Aldrich) in a 10 mL volumetric flask. Following the addition of 2 mL of sodium carbonate (7.5%, Sigma-Aldrich) to the mixture, TPC was determined after a 60 min incubation at 37 °C. After incubation, the absorbance versus the prepared blank was measured at 765 nm. The standard reference curve of gallic acid (Sigma-Aldrich) was obtained for the following concentrations: 1, 2, 4, 6, 8, and 10 mg/mL. TPC was determined and expressed as milligram gallic acid equivalents (mg GAE/100 g) using the standard curve as a reference. All measurements were performed thrice.

### 2.5. DPPH Free Radical Scavenging Ability

The free radical scavenging assay of DPPH was used to evaluate the antioxidant properties of the essential oils. The measurement was based on the method described in a previous study [39]. First, 0.1 mL of essential oils was added to 1.9 mL of a 0.3 mM DPPH solution (Sigma-Aldrich) at different concentrations (1, 2, 4, 6, 8, and 10 mg/mL). The solution was mixed vigorously and allowed to stand at room temperature for 30 min in the dark. Then, its absorbance was measured using a spectrophotometer at 517 nm, and ethanol (SAMCHUN) was used as a blank and ascorbic acid as a positive control. A simple regression analysis was performed to determine EC_50_. All experiments were repeated three times.

### 2.6. ABTS Free Radical Scavenging Assay

The scavenging activity of ABTS^+^ was investigated to measure the radical scavenging activity. The ABTS radical scavenging activity of the essential oils was determined following the procedure described by Re et al. [40]. ABTS^+^ was generated by reacting an ABTS^+^ aqueous solution (7 mM, Sigma-Aldrich) with a potassium persulfate solution (2.45 mM, SAMCHUN) at 37 °C for 16 h and adjusting the absorbance at 734 nm to 0.7 ± 0.02 using ethanol. Then, 0.2 mL of the essential oils at different concentrations was added to 2 mL of the ABTS^+^ solution. Then, the absorbance was measured at 734 nm after 30 min. Ascorbic acid (Sigma-Aldrich) was used as a positive control. A simple regression analysis was conducted to determine the EC_50_ value. All measurements were performed thrice.

### 2.7. Determination of Ferric-Reducing Antioxidant Potential

This method is based on the ability of the sample to reduce Fe^3+^ to Fe^2+^ ions. The ferric-reducing antioxidant potential (FRAP) assay was performed following a procedure described in a previous study with some modifications [41]. The working FRAP reagent (Sigma-Aldrich) was prepared by mixing 300 mM acetate buffer (pH 3.6), 10 mM 2,4,6-tri-2-pyridyl-1,3,5-triazin (TPTZ, Supelco, Bellefonte, PA, USA) in 40 mM HCl (SAMCHUN), and 20 mM ferric chloride (Sigma-Aldrich) at a ratio of 10:1:1. The FRAP reagent was prepared immediately before. Then, 4.5 mL aliquot of the FRAP reagent was mixed with 150 μL of essential oils of various concentrations. The mixture was vigorously shaken for 30 s and kept in the dark at 37 °C for 1 h. Thereafter, the absorbance was measured at 593 nm using ethanol as the blank solution. The obtained absorbance was expressed as micromole FeSO_4_·H_2_O equivalent/milligram essential oils relative to the values from a standard curve obtained using Fe^2+^ solutions of known concentrations. All the measurements were performed thrice.

### 2.8. Statistical Analysis

All the assays were conducted thrice, and the results are expressed as the mean ± standard deviation. An analysis of variance was performed to check the significant differences using the Statistical Package for the Social Sciences (SPSS ver. 24.0; IBM, Seoul, Republic of Korea) program. Tukey HSD tests were conducted to compare the various groups. Differences from the control were considered significant when the *p*-values were lower than 0.05.

## 3. Results

### 3.1. Chemical Composition of the Essential Oils

The chemical composition of the essential oils, as determined by GC–MS, is listed in Table 2 only for the components with a content of 0.5% or more. The 67 compounds were identified, accounting for 99.1–99.9% of the total oil. The essential oils of 21 citrus cultivars contained large amounts of monoterpene hydrocarbon (83.7–99.0%) with very small amounts of oxygenated monoterpene (0.5–13.4%), sesquiterpene hydrocarbon (0.02–1.5%), and oxygenated sesquiterpene (0.00–1.2%). Oxygenated sesquiterpene was not detected in the essential oils extracted from BY, KA, PO, PU, SH, ST, and YN. A minimum of 18 compounds were identified in the essential oil of PU, whereas that of PL contained a maximum of 39 compounds. The essential oils of all the citrus cultivar samples contained nine components in common (α-pinene, d-limonene, *trans*-β-ocimene, γ-terpinene, terpinolene, linalool, β-terpineol, terpinene-4-ol, and α-terpineol). Among them, d-limonene (50.9–97.2%), γ-terpinene (0.02–27.3%), α-terpineol (0.2–5.9%), and α-pinene (0.2–2.0%) were the major components.

### 3.2. Total Phenolic Contents

Phenolic compounds are related to several biological roles, such as free radical scavenging [42]. The TPCs of the essential oils were measured using the Folin–Ciocalteu method and are expressed as milligrams of gallic acid equivalents (GAE) per 100 g of the sample (mg GAE/100 g).

As shown in Table 3, the TPCs of the essential oils were significantly different. The essential oil of YU contained the highest amount of phenolics (360.04 ± 24.75 mg GAE/100 g), followed by PL (339.42 ± 31.14 mg GAE/100 g), TS (327.05 ± 14.29 mg GAE/100 g), ST (322.92 ± 21.43 mg GAE/100 g), and PU (318.80 ± 18.90 mg GAE/100 g), in that order. The least phenol was observed in the essential oil of KA (219.82 ± 14.29 mg GAE/100 g).

### 3.3. DPPH Radical Scavenging

The results of the DPPH inhibition assay are listed in Table 3. All the essential oils reduced the pink free radical to a yellow diphenyl picrylhydrazine, indicating that the essential oils exhibited DPPH radical scavenging activity.

Among the citrus cultivar samples, the essential oil of ST showed the highest radical scavenging activity with an IC_50_ of 86.17 ± 4.87 mg/mL, followed by SH (IC_50_ = 148.60 ± 8.20 mg/mL) and DA (IC_50_ = 165.86 ± 12.49 mg/mL). However, the radical scavenging activity of the essential oils was lower than that of the ascorbic acid.

### 3.4. ABTS Radical Scavenging Assay

Table 4 lists the comparative ABTS radical scavenging activity of the essential oils from citrus species and ascorbic acid.

Among the essential oils, those from ST showed the highest scavenging activity (IC_50_ = 0.16 ± 0.06 mg/mL), followed by those from YU (IC_50_ = 0.28 ± 0.03 mg/mL) and DA (IC_50_ = 0.52 ± 0.18 mg/mL). Comparing values with those of the standard antioxidant (ascorbic acid; IC_50_ = 0.01 ± 0.00 mg/mL), we found that the essential oils from citrus cultivars exhibited significantly lower scavenging activity than the standard antioxidant.

### 3.5. FRAP Assay

The antioxidant efficiency of the extracted essential oils was evaluated using the FRAP method, with reference to the reaction formula given by an Fe^2+^ solution of known concentration. The reducing powers of the essential oils are listed in Table 5.

Among the essential oils, those from PO (2302.55 ± 237.26 mg/100 g) showed the highest reducing power, followed by those from ST (2213.12 ± 35.54 mg/100 g) and MW (2071.92 ± 155.54 mg/100 g), and the essential oil from LI showed the lowest reducing power.

**Figure 1:**
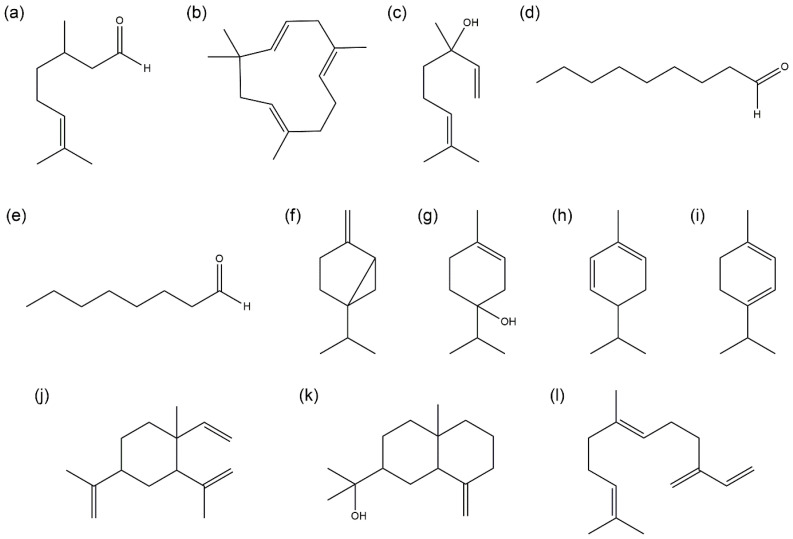
Chemical structures of the active compounds: (**a**) citronellal, (**b**) humulene, (**c**) linalool, (**d**) nonanal, (**e**) octanal, (**f**) sabinene, (**g**) terpinene-4-ol, (**h**) α-phellandrene, (**i**) α-terpinene, (**j**) β-elemene, (**k**) β-eudesmol, (**l**) β-farnesene.

Among the essential oils, the oil from ST showed the strongest antioxidant activity, followed by those from TS, YU, and DA. To identify the constituents of the oils contributing to their antioxidant activities, candidate constituents were selected by statistically analyzing the composition and antioxidant activities of citrus cultivars using the SIMCA 17 program. The antioxidant activities of these candidate constituents were evaluated. The components of citrus essential oils with a superior antioxidant activity were statistically analyzed to investigate the candidate substances that contributed to the antioxidant activity. Based on the obtained results, 12 single compounds (citronellal, humulene, linalool, nonanal, octanal, sabinene, terpinene-4-ol, α-phellandrene, α-terpinene, β-elemene, β-eudesmol, and β-farnesene) were selected as active antioxidant components (Figure 1). Structural features such as aldehyde, hydroxyl, and conjugated diene were observed in the 12 single compounds.

The antioxidant activities of the 12 single compounds were determined using DPPH, ABTS, and FRAP assays. The DPPH and ABTS free radical scavenging abilities are listed in Table 6.

The IC_50_ of the 12 compounds ranged from 204.30 ± 31.29 mg/mL (α-phellandrene) to 2026.76 ± 237.68 mg/mL (Terpinen-4-ol). α-Phellandrene exhibited the highest DPPH radical scavenging activity, followed by α-terpinene (IC_50_ = 242.30 ± 30.28 mg/mL), humulene (IC_50_ = 376.77 ± 4.22 mg/mL)*,* β-farnesene (IC_50_ = 394.52 ± 58.13 mg/mL), β-elemene (IC_50_ = 451.89 ± 9.04 mg/mL), and citronellal (IC_50_ = 479.52 ± 9.13 mg/mL), in that order.

In the ABTS assay, α-terpinene was the most active constituent based on its IC_50_ (0.21 ± 0.10 mg/mL), followed by humulene (IC_50_ = 2.69 ± 0.19 mg/mL)*,* citronellal (IC_50_ = 5.29 ± 1.83 mg/mL), α-phellandrene (IC_50_ = 5.32 ± 0.05 mg/mL), β-farnesene (IC_50_ = 7.43 ± 0.56 mg/mL), and β-elemene (IC_50_ = 7.83 ± 1.09 mg/mL), in that order. However, all the single compounds showed weak activities compared to those of ascorbic acid. Terpinene-4-ol and nonanal showed the lowest activities, with an IC_50_ of 36.05 ± 4.79 mg/mL and 30.19 ± 10.01 mg/mL, respectively.

The FRAP assay measured the total antioxidant activity of the oil samples, and the results are listed in Table 7. The antioxidant activities are expressed as milligrams of antioxidants having a ferric-reducing ability equivalent to that of 100 g of FeSO_4_. The ferric-reducing capacity ranged from 587.57 ± 16.30 to 6519.82 ± 99.18 mg/100 g. α-Terpinene showed the highest reducing power (6519.82 ± 99.18 mg/100 g), followed by α-phellandrene (4015.81 ± 129.41 mg/100 g), β-farnesene (3841.66 ± 66.73 mg/100 g), β-elemene (3625.15 ± 282.88 mg/100 g), and humulene (3418.06 ± 139.31 mg/100 g).

These results indicate that α-terpinene and α-phellandrene contributed most to the antioxidant activity of the essential oil from citrus cultivars. Based on these results, it is estimated that the conjugated diene, which is the common feature of the two components, is the structural feature contributing to antioxidant activities.

## 4. Discussion

Essential oils from citrus cultivars have been extensively studied and are widely used in the food, cosmetic, and pharmaceutical industries. The essential oils from the peels of citrus cultivars are widely used as flavoring agents, as well as cosmetic and medicinal ingredients due to their superior antioxidant properties [43]. The essential oils from citrus cultivars result in a much greater risk than those used in cosmetics, as they directly affect the human body through oral exposure [44]. However, according to the U.S. Food and Drug Administration (FDA), they are recognized as safe as flavoring agents. Additionally, lime, lemon, grapefruit, and orange oils are approved for use as flavoring agents in the United States Pharmacopeia (USP) Food Chemicals Codex [44]. The essential oils used in cosmetics have the potential for irritation and sensitization from dermal exposure. Therefore, the essential oils from cultivars are considered safe under these limitations, and caution is advised when using them [45]. According to the International Cosmetic Ingredient Dictionary and Handbook, citrus oils function only as a fragrance ingredient [46].

In this study, we extracted and characterized essential oils from 21 citrus cultivar samples collected from Jeju Island. The essential oils were extracted via hydrodistillation, and their chemical composition was analyzed by GC–MS. D-Limonene (50.88–97.19%) was the most abundant monoterpene hydrocarbon in all the essential oil samples (Table 2), and all essential oils showed antioxidant activity via different mechanisms, as revealed by different antioxidant assays. Among them, oil from [(*C. unshiu* × *C. sinensis*) × *C. reticulata*] × *C. reticulata* showed the highest antioxidant potential (Table 3, Table 4 and Table 5). Furthermore, we selected 12 single compounds that contributed the most to the antioxidant activity of the essential oils based on their chemical composition. Among them, α-phellandrene and α-terpinene showed high antioxidant activity (Table 6 and Table 7).

The most abundant constituent of the essential oils from the citrus species was d-limonene. Limonene is a common constituent of essential oils from aromatic plants, and it serves as a precursor for the biosynthesis of other monoterpenes [47]. In previous studies, limonene has been investigated for various pharmacological applications. Limonene exhibits anti-inflammatory, antimicrobial, anticancer, and antinociceptive activities, all of which are attributed to its structural characteristics [48,49,50,51]. Limonene comprises two isoprene units and has two double bonds. Compounds made up of the isoprene structure possess antioxidant properties [52]. Therefore, essential oils from citrus cultivars exhibit antioxidant activity owing to their volatile constituents and structural characteristics. This study shows that α-phellandrene and α-terpinene had the most superior antioxidant activity. The structural characteristics of the two components are conjugated diene, which is different from limonene. Since limonene is the main component of citrus essential oil, it is necessary to compare the antioxidant activity of limonene and conjugated diene structural components by considering the composition ratio in the essential oil.

Phenolics are secondary metabolites in plants exhibiting physiological activities, such as antioxidant activity [53]. In addition, they contain phenolic hydroxyl, which can bind to proteins and macromolecules [54]. These phenolic compounds are free radical scavengers that inhibit the initiation of lipid oxidation or interfere with its propagation, thereby reducing the formation of volatile decomposition products that cause rancidity, such as aldehydes and ketones [55]. Thus, their TPC is related to their antioxidant capacity [56]. Among the 21 citrus cultivar samples, the essential oil from *C. junos* showed the highest amount of phenolics. However, the essential oil from [(*C. unshiu* × *C. sinensis*) × *C. reticulata*] × *C. reticulata* showed the highest radical scavenging activity. For all essential oils, the antioxidant activities did not vary with their TPC. The antioxidant activities of the oils can be attributed to other types of metabolites, such as flavonoids [57,58]. This is consistent with previous reports, indicating that TPC is not related to the antioxidant activity of oils [59,60].

Several studies have reported that the essential oils from *C. reticulata*, *C. limon*, and *C. paradisi* have a higher radical scavenging ability than those from citrus cultivars [33,35,61,62,63]. However, in this study, the essential oil from [(*C. unshiu* × *C. sinensis*) × *C. reticulata*] × *C. reticulata*, *C. maxima*, and (*C. unshiu* × *C. sinensis*) × *C. unshiu* showed a higher antioxidant activity than that from *C. reticulata*, *C. limon*, and *C. paradisi*. To date, the antioxidant activity of the oils from [(*C. unshiu* × *C. sinensis*) × *C. reticulata*] × *C. reticulata*, *C. maxima*, and (*C. unshiu* × *C. sinensis*) × *C. unshiu* has not been investigated; only a limited number of species have been investigated. However, in this study, we investigate the antioxidant activities of various citrus cultivars. Thus, this study serves as a good basis for the more efficient use of citrus cultivars.

Citrus cultivars are derived from various citrus species because different species easily hybridize [64]. However, herein, the chemical composition of the essential oils was similar. Therefore, the major constituents of the oil from [(*C. unshiu* × *C. sinensis*) × *C. reticulata*] × *C. reticulata* contributing to the antioxidant activities were investigated. D-Limonene (50.9–97.2%) was the most abundant monoterpene hydrocarbon in all essential oils (Table 2). However, d-limonene had no significant effect on the antioxidant activity of the oils. According to the study by Choi et al., citrus oils rich in γ-terpinene and terpinolene exhibit a high radical scavenging ability [65]. In this study, we found that α-terpinene and α-phellandrene contribute the most to the antioxidant activity of the essential oils. However, these two compounds are minor constituents of the essential oil from [(*C. unshiu* × *C. sinensis*) × *C. reticulata*] × *C. reticulata*. Minor compounds play a significant role in the antioxidant activity and have synergistic effects [66]. Badalamenti reported that minor compounds contribute to the antioxidant activity of *C. aurantium* [67]. Among the combinations of oils studied herein, a mixture of *C. aurantium* “Canaliculata” and “Bizzaria” (1:1 = *v*/*v*) showed a promising antioxidant activity. The sample exhibited a relatively low IC_50_ with a promising DPPH, ABTS, and β-carotene radical scavenging potential. These results show the importance of minor compounds in the antioxidant activity of essential oil samples.

## 5. Conclusions

As interest in health increases, the demand for natural antioxidants is steadily increasing. For this reason, many studies have been conducted to evaluate the antioxidant effect of essential oils extracted from plants. Among them, citrus cultivars are attracting attention because they contain various antioxidant compounds such as vitamins, phenolic compounds, terpenoids, etc. However, these studies are focused only on some citrus species; therefore, this study evaluates the antioxidant effect of essential oils extracted from 21 citrus cultivars. Among them, the essential oil from [(*C. unshiu* × *C. sinensis*) × *C. reticulata*] × *C. reticulata* had a superior antioxidant effect, and α-phellandrene and α-terpinene were found to be active constituents of the oil. Considering that the two single compounds are minor components in the essential oil from [(*C. unshiu* × *C. sinensis*) × *C. reticulata*] × *C. reticulata*, it is necessary to evaluate the antioxidant effect by applying different concentrations according to the proportion in the total essential oil. In addition, since single compounds can exert a synergistic effect and exhibit antioxidant effects, research is also needed to evaluate the synergistic effect of single compounds with a superior activity. The citrus species that are prone to interspecific hybridization differ depending on the hybridized species, and the essential oil components and activities differ depending on the citrus variety. By comparing the results of this study with the phylogenetic classification study of citrus species, it will be possible to search for phylogenies with a superior antioxidant activity. These studies could crossbreed citrus cultivars with a superior antioxidant activity. In addition, the citrus essential oils as natural antioxidants will be scientifically proven, allowing them to be widely used.

## Figures and Tables

**Table 1:** Summary of cultivar information and citrus peels.

No.	Sample Name	Common Name	Abbreviation	Voucher Specimen No.
1	*Citrus japonica* Thunb.	Kumquat	KU	WTFRC10032742
2	*Citrus junos* Siebold ex Tanaka	Yuzu	YU	WTFRC10032743
3	*Citrus limon* (L.) Osbeck ‘Lisbon’	Lisbon lemon	LI	WTFRC10033803
4	*Citrus maxima* (Burm.) Merr.	Dangyuja	DA	WTFRC10032725
5	*Citrus maxima* (Burm.) Merr. ^a^	Pomelo	PU	WTFRC10032744
6	*Citrus medica* L. ^b^	Buddha’s hand	FC	WTFRC10033804
7	*Citrus paradisi* Macfad. ‘Redblush’	Grapefruit	RU	WTFRC10032741
8	*Citrus platymamma* hort. ex Tanaka	Byungkyul	BY	WTFRC10032726
9	*Citrus reticulata* Blanco ^c^	Miyagawa Satsuma	MW	WTFRC10032727
10	*Citrus reticulata* Blanco ‘Ponkan’	Ponkan	PO	WTFRC10032734
11	*Citrus reticulata* Blanco ^d^	Satsuma	SM	WTFRC10032740
12	*Citrus sinensis* (L.) Osbeck ‘Navel’	Orange	YN	WTFRC10032732
13	*Citrus sunki* (Hayata) Yu.Tanaka	Jinkyul	JI	WTFRC10032733
14	*Citrus* × *aurantium* L. ^e^	Amanatsu	NA	WTFRC10032737
15	*Citrus* × *aurantium* L. ^f^	Kamja	KA	WTFRC10032735
16	*Citrus* × *aurantium* L. ^g^	Seminole	SE	WTFRC10032729
17	*Citrus* × *latifolia* (Yu.Tanaka) Yu. Tanaka	Persian lime	PL	WTFRC10032736
18	*Citrus unshiu* × *Citrus sinensis*	Kiyomi	KY	WTFRC10032739
19	(*Citrus unshiu* × *Citrus sinensis*) × *Citrus reticulata*	Shiranui	SH	WTFRC10032728
20	(*Citrus unshiu* × *Citrus sinensis*) × *Citrus unshiu*	Tsunokaori	TS	WTFRC10032731
21	[(*Citrus unshiu* × *Citrus sinensis*) × *Citrus reticulata*] × *Citrus reticulata*	Setoka	ST	WTFRC10032730

^a^ Synonym of *C. grandis* (L.) Osbeck, ^b^ synonym of *C. medica* var. sarcodactylus (Siebold ex Hoola van Nooten) Swingle, ^c^ synonym of *C. unshiu* (Yu.Tanaka ex Swingle) Marcow, “Miyagawa-wase”, ^d^ synonym of *C. unshiu* (Yu.Tanaka ex Swingle) Marcow, ^e^ synonym of *C.* × *natsudaidai* (Yu.Tanaka) Hayata, ^f^ synonym of *C.* × *benikoji* Yu.Tanaka, ^g^ synonym of *C.* × *tangelo* J.W.Ingram and H.E.Moore.

**Table 2:** Chemical composition of the essential oils from the 21 citrus cultivar samples.

KI ^a^	Compound Name	KU	YU	LI	DA	PU	FC	RU	BY	MW	PO	SM	YN	JI	NA	KA	SE	PL	KY	SH	TS	ST
920	α-Thujene		0.2	0.1			0.6			0.1	0.1	0.1			^tr^		^tr^	0.2			0.1	
926	α-Pinene	0.3	1.1	1.1	0.3	0.2	2.0	0.4	0.3	0.6	0.7	0.7	0.3	0.5	0.5	0.4	0.6	1.6	0.3	0.4	0.6	0.5
965	Sabinene		^tr^	0.1			0.1	0.3	0.1	^tr^	0.1	^tr^	^tr^	0.1		^tr^		0.2	^tr^	0.2	0.2	0.6
970	β-Pinene		0.5	6.0		0.2	1.6	0.1	0.9	0.3	0.3	0.3		2.0	0.2	0.9	0.1	7.6		^tr^	0.3	0.4
986	β-Myrcene	1.3	1.3	1.0	21.6	28.1		1.3	19.5	1.2	1.2	1.3	1.3	1.2	1.1	1.2	1.3	0.8	1.2	1.4	1.2	1.1
1024	α-Terpinene		0.4	0.3			0.5	0.3	^tr^	0.1	0.1	0.1	0.1	0.1	0.1	^tr^	0.1	0.6	0.1	0.2	0.2	0.4
1034	*m*-Cymene		0.6	1.3		^tr^	0.8	^tr^		0.3	0.1	0.4	^tr^		0.2		0.1	1.9			1.3	0.1
1041	D-Limonene	97.2	78.0	69.0	76.3	68.8	59.2	93.5	77.1	90.6	90.5	89.3	95.7	92.4	90.4	95.2	92.0	50.9	96.5	94.2	89.2	91.1
1047	*cis*-β-Ocimene			^tr^	0.1		1.2		0.1					0.1		^tr^		^tr^		0.1		
1058	*trans*-β-Ocimene	^tr^	0.3	0.1	0.4	0.2	1.8	0.4	0.5	0.1	0.1	0.1	^tr^	0.4	0.2	0.1	0.3	0.1	0.1	0.4	0.2	0.1
1068	γ-Terpinene	^tr^	11.5	9.7	^tr^	0.1	27.3	0.7	0.1	4.6	5.1	5.5	0.2	0.1	5.0	0.1	3.4	17.6	0.1	0.4	3.9	0.8
1090	Terpinolene	^tr^	0.7	1.0	0.1	0.2	1.3	0.3	0.1	0.3	0.3	0.3	0.1	0.1	0.4	0.2	0.2	1.9	0.1	0.1	0.3	0.2
1099	Linalool	0.1	2.0	0.4	0.1	0.2	0.1	0.2	0.4	0.4	0.7	0.1	0.4	0.7	0.2	0.6	0.3	0.7	0.1	0.5	0.3	1.0
1179	Terpinen-4-ol	^tr^	0.3	1.2	^tr^	0.1	0.5	1.1	0.1	0.1	0.2	0.1	0.3	0.2	0.2	0.1	0.1	1.7	0.2	0.7	0.7	1.8
1194	α-Terpineol	0.3	1.0	3.3	0.2	0.4	0.7	0.6	0.3	0.4	0.3	0.3	0.6	0.5	0.8	0.6	1.0	5.9	0.5	0.5	0.4	0.5
1243	(*Z*)-Citral			1.4		0.1	0.3											1.7				
1273	(*E*)-Citral			2.2		0.1	0.3											2.4				
1362	Neryl acetate			0.3											0.1		^tr^	0.9	0.1		0.1	
1395	β-Elemene	^tr^	0.1		^tr^			^tr^	^tr^	0.5	^tr^	0.7	^tr^	1.0	0.1	0.1	^tr^	0.1	^tr^		0.1	0.1
1513	β-Bisabolene	^tr^		0.1			0.1		0.1	0.1					^tr^			0.5				
Monoterpene hydrocarbons		99.0	95.1	90.0	98.9	97.9	96.4	97.3	98.6	98.0	98.4	98.2	97.9	96.8	98.1	98.1	98.1	83.7	98.4	97.5	97.4	95.4
Oxygenated monoterpenes		0.5	3.5	9.1	0.6	1.2	1.7	2.4	0.9	1.0	1.3	0.5	1.6	1.5	1.4	1.6	1.6	13.4	1.1	2.2	2.0	4.0
Sesquiterpene hydrocarbons		0.2	0.9	0.3	0.2	^tr^	0.5	0.2	0.2	0.8	^tr^	1.1	0.4	1.3	0.2	0.1	0.1	1.5	0.1	0.1	0.2	0.1
Oxygenated sesquiterpenes		0.1	0.2	0.5	^tr^	^tr^	^tr^	^tr^	^tr^	^tr^	^tr^	^tr^	^tr^	0.1	0.1	^tr^	^tr^	1.2	0.1	^tr^	0.1	^tr^
Unknown compounds		0.2	0.4	0.2	0.3	0.9	1.4	0.2	0.3	0.1	0.2	0.2	0.1	0.3	0.2	0.2	0.1	0.3	0.4	0.2	0.3	0.5

^a^ The Kovats retention index was experimentally determined using a VF-5MS column with a homologous series of C_8_–C_30_ alkanes. ^tr^ trace amount (<0.05%).

**Table 3:** Total polyphenol contents and DPPH radical scavenging activity of the essential oils of the citrus cultivar samples.

Sample	TPC (mg GAE/100 g)	DPPH (IC_50_, mg/mL)
KU	244.56 ± 14.29 *	2215.52 ± 203.67 *
YU	360.04 ± 24.75 *	462.07 ± 33.48 *
LI	306.43 ± 18.90 *	1612.08 ± 2.16 *
DA	310.55 ± 12.37 *	165.86 ± 12.49 *
PU	277.56 ± 14.29 *	1719.85 ± 196.09 *
FC	318.80 ± 18.90 *	1185.47 ± 150.95 *
RU	265.19 ± 7.14 *	536.91 ± 54.30 *
BY	281.68 ± 37.80 *	304.25 ± 20.41 *
MW	265.19 ± 18.90 *	305.44 ± 28.38 *
PO	314.68 ± 43.45 *	282.74 ± 15.05 *
SM	289.93 ± 7.14 *	262.50 ± 10.53 *
YN	265.19 ± 14.29 *	333.29 ± 48.96 *
JI	289.93 ± 18.90 *	203.75 ± 63.39 *
NA	256.94 ± 18.90 *	1059.67 ± 0.00 *
KA	219.82 ± 14.29 *	515.45 ± 45.22 *
SE	269.31 ± 31.14 *	432.89 ± 65.86 *
PL	339.42 ± 31.14 *	3025.67 ± 153.06 *
KY	223.94 ± 12.37 *	704.05 ± 40.37 *
SH	314.68 ± 18.90 *	148.60 ± 8.20 *
TS	327.05 ± 14.29 *	221.56 ± 16.53 *
ST	322.92 ± 21.43 *	86.17 ± 4.87 *

Data are presented as the mean ± standard deviation (*n* = 3). * Represents *p*-values lower than 0.05.

**Table 4:** ABTS radical scavenging activity of the essential oils from the 21 citrus cultivar samples.

Sample	ABTS (IC_50_, mg/mL)
KU	12.08 ± 2.13 *
YU	0.28 ± 0.03 *
LI	4.98 ± 0.48 *
DA	0.52 ± 0.18 *
PU	4.83 ± 1.00 *
FC	2.79 ± 0.20 *
RU	2.44 ± 0.09 *
BY	1.34 ± 0.28 *
MW	1.04 ± 0.29 *
PO	0.60 ± 0.05 *
SM	0.84 ± 0.10 *
YN	1.02 ± 0.07 *
JI	0.66 ± 0.04 *
NA	3.36 ± 0.17 *
KA	4.49 ± 0.30 *
SE	0.80 ± 0.12 *
PL	5.16 ± 0.69 *
KY	6.04 ± 0.28 *
SH	0.72 ± 0.16 *
TS	0.56 ± 0.08 *
ST	0.16 ± 0.06
Ascorbic acid	0.01 ± 0.00 *

Data are presented as the mean ± standard deviation (*n* = 3). * Represents *p*-values lower than 0.05.

**Table 5:** Ferric-reducing activity of the essential oils from the 21 citrus cultivars.

Sample	FRAP (mg/100 g)	Sample	FRAP (mg/100 g)
BY	1624.78 ± 71.07 *	PO	2302.55 ± 237.26 *
DA	1685.96 ± 74.72 *	PU	1267.06 ± 77.77 *
FC	1620.07 ± 29.39 *	RU	1690.67 ± 21.57 *
JI	1808.34 ± 58.79 *	SE	1737.74 ± 63.67 *
KA	1549.47 ± 80.29 *	SH	1770.69 ± 37.36 *
KU	1248.23 ± 120.64 *	SM	1676.55 ± 94.02 *
KY	1257.65 ± 91.87 *	ST	2213.12 ± 35.54 *
LI	425.49 ± 0.00 *	TS	1690.67 ± 158.71 *
MW	2071.92 ± 155.54 *	YN	2015.44 ± 45.39 *
NA	815.21 ± 29.39 *	YU	1403.56 ± 64.71 *
PL	1290.60 ± 64.71 *		

Data are presented as the mean ± standard deviation (*n* = 3). * Represents *p*-values lower than 0.05.

**Table 6:** Antioxidant activity of single compounds as determined using DPPH and ABTS assays.

Compound Name	DPPH (IC_50_, mg/mL)	ABTS (IC_50_, mg/mL)
Citronellal	479.52 ± 9.13	5.29 ± 1.83 *
Humulene	376.77 ± 4.22	2.69 ± 0.19 **
Linalool	813.71 ± 13.40	15.13 ± 4.95 *
Nonanal	1020.32 ± 410.20	30.19 ± 10.01
Octanal	935.06 ± 35.13	12.59 ± 1.77 *
Sabinene	823.90 ± 0.00	15.70 ± 3.12 **
Terpinen-4-ol	2026.76 ± 237.68	36.05 ± 4.79 **
α-Phellandrene	204.30 ± 31.29	5.32 ± 0.05 **
α-Terpinene	242.30 ± 30.28	0.21 ± 0.10 *
β-Elemene	451.89 ± 9.04	7.83 ± 1.09 **
β-Eudesmol	595.26 ± 40.21	12.25 ± 0.18 **
β-Farnesene	394.52 ± 58.13	7.43 ± 0.56 **
Ascorbic acid	2.45 ± 0.77 *	0.05 ± 0.01 **

Data are presented as the mean ± standard deviation (*n* = 3). ** Represents *p*-values lower than 0.01, and * represents *p*-values lower than 0.05.

**Table 7:** Antioxidant activity of single compounds, as determined using the FRAP assay.

Compound Name	FRAP (mg/100 g)
Citronellal	843.45 ± 8.15 **
Humulene	3418.06 ± 139.31 **
Linalool	852.87 ± 14.12 **
Nonanal	587.57 ± 16.30 **
Octanal	1168.22 ± 29.39 **
Sabinene	2180.18 ± 101.82 **
Terpinen-4-ol	829.33 ± 8.15 **
α-Phellandrene	4015.81 ± 129.41 **
α-Terpinene	6519.82 ± 99.18 **
β-Elemene	3625.15 ± 282.88 **
β-Eudesmol	1224.70 ± 57.07 **
β-Farnesene	3841.66 ± 66.73 **

Data are presented as the mean ± standard deviation (*n* = 3). ** Represents *p*-values lower than 0.01.

## Data Availability

Data is contained within the article.

## Abbreviations

ANOVA	Analysis of variance
EI	Electron ionization
FRAP	Ferric-reducing antioxidant potential
KI	Kovats retention index
ROS	Reactive oxygen species
RSM	Response surface methodology
SPSS	Statistical Package for the Social Sciences
TPC	Total phenolic content
